# Practical Departments

**Published:** 1905-07-29

**Authors:** 


### PRACTICAL DEPARTMENTS.

### FIRE-RESISTING DOORS.

The fireproof doors manufactured by Messrs. Mather and Platt, of Salford Ironworks, Manchester, are of a novel design. They are constructed of three or four thicknesses of well-seasoned pine boards, planed, tongued and grooved, nailed together with wrought-iron nails driven flush, and clenched on the other side, the door proper being built up of wood. Outside the wood sheets of tinned steel are fixed, in such a manner that, it is claimed, they are free to expand under heat, but cannot become detached, and therefore exclude the air from the wood inside. The *rationale* of the fire-resisting arrangement is, the presence of the steel sheets prevents the air from reaching the wood as long as they are intact, and therefore the wood cannot burn, in the ordinary sense. Combustion cannot take place, as there is no oxygen for the carbon of the wood to combine with. The wood becomes charred, partly carbonised, if the door is exposed to heat for long; but it is claimed, it continues to fulfil its functions as a door, and to perform important offices, such as excluding air from portions of the building, and so restricting the area of the fire. The doors are made for use in any form—horizontal, sliding, hinged, folding, double sliding, or vertical sliding. Window-shutters, trap-doors, and other applications of the principle are also made. The makers point out that their doors remain in place and perform their offices, when iron or steel doors give way very quickly, the heat expanding them and causing them to burst their holdings.

### AIRLIGHT.

The British American Safety Airlight Company, of 66 Victoria Street, London, S.W., are introducing an apparatus into this country with the above name, the principal feature of which, by the published descriptions, appears to be the fact, claimed by the introducers, that it burns 95 per cent. of air, and 5 per cent. of petrol or gasoline vapour. The real speciality seems to us to be the adaptation of the principle of the Welsbach mantle to the consumption of petroleum. Gas, when consumed in the ordinary burner is very wasteful and gives a yellow light, the reason being that the light emanates from the hot particles of carbon on the edge of the flame that are on their way to the outer atmosphere and are already partially cooled. The heat is expended upon the atmosphere of the room, but without giving out the full light possible in the process. In the Welsbach mantle, the carbon particles are arrested by the mantle, and the latter being raised to a very high temperature, very much more light is given out for a given consumption of gas. In the ordinary oil lamp, as is well known, it is not the oil that is burnt, it is the vapour of the oil which is liberated by the heat of the lamp itself; but the same trouble arises as in the gas burner, the light is given by the particles of incandescent carbon on the edge of the flame, which are already partly cooled. So far no attempt has been successful to adapt the mantle to the oil lamp and, as we understand the matter, the Airlight is intended to fill this gap. The apparatus consists of a copper cylinder, forming a reservoir for the petrol or gasoline; a generator, consisting of a pipe surrounded by two ring burners, called by the inventor sub-flames; a mixer, consisting of a smaller tube, to which another larger tube is connected, leading to the atmosphere; a governor and pipe leading to the burners, which are mantles on the Welsbach principle, specially designed for this purpose and very much larger than the mantle we are familiar with. The generator corresponds to the carburrettor of a petrol engine. It is in communication with the reservoir on the one hand and with the mixer on the other, and its office is to liberate vapour from the petrol by the application of heat, the heat being supplied by the ring burners, which are also fed from the generator. In the mixer the air, which is drawn in by the draught created, mixes with the vapour and the two pass on to the burner, where the petrol vapour is split up in the usual way into its components, principally carbon and hydrogen, and where combustion takes place, the mantle being heated up as in the case of gas. The governor controls the supply of petrol to the generator, in accordance with the consumption of the burners. One generator and reservoir is arranged to supply a number of lamps, the lamps being connected to the generator by pipes in the usual way; but as the mixture contains such a large proportion of air, the pipes have to be made very much larger than is usual with gas. For one lamp the pipe required is stated to be 1 inch in diameter, for six lamps $1\frac{1}{2}$ inch, and for 12 lamps 2 inches. The apparatus is made in the following sizes: For working any number of lamps up to eight, for 14, 22, and 28. It is claimed that the light given by this system is very economical, but the economy appears to be based very largely upon the power of the light given by each mantle, which is stated to be equal to six ordinary Welsbach burners, burning ordinary coal gas. The pipe used is made of tinned or galvanised sheet iron, something on the pattern of speaking-tubes, it being claimed that, the pressure being very low, this pipe is suitable.

